# High molecular weight adiponectin inhibits vascular calcification in renal allograft recipients

**DOI:** 10.1371/journal.pone.0195066

**Published:** 2018-05-02

**Authors:** Kanae Nomura-Nakayama, Hiroki Adachi, Nobuhiko Miyatake, Norifumi Hayashi, Keiji Fujimoto, Hideki Yamaya, Hitoshi Yokoyama

**Affiliations:** Department of Nephrology, Kanazawa Medical University School of Medicine, Uchinada, Ishikawa, Japan; Centro Cardiologico Monzino, ITALY

## Abstract

**Background:**

Adiponectin (ADPN) prevents the development/recurrence of cardiovascular events via its anti-atherogenic effects. However, few long-term studies have examined the changes in serum ADPN levels and arterial calcification seen in renal allograft recipients.

**Subjects and methods:**

The effects of the serum ADPN level on arterial calcification were examined in 51 Japanese renal allograft recipients. Abdominal aorta calcification was evaluated on computed tomography using the aortic calcification area index (ACAI). The change in the ACAI and serum high-molecular-weight (HMW)—ADPN fractions were studied over an 8-year period. The arterial expression of ADPN, ADPN receptors (AdipoR)1 and 2, and T-cadherin (cadherin-13) were also examined by immunohistochemistry.

**Results:**

1. The change in the ACAI were grouped into quartiles and compared with the alterations in the serum levels of each ADPN fraction over an 8-year period. The change in the ACAI was much lower in the patients with highly elevated HMW-ADPN levels.
2. Multiple regression analysis demonstrated that an advanced age at transplant and a history of cardiovascular complications were associated with an increased change in the ACAI, while higher HMW-ADPN concentrations were associated with improvements in the ACAI. Serum HDL-C level was also identified as a positive factor to increase serum HMW-ADPN level.
3. In immunohistochemical examinations, ADPN was detected on CD31-positive arterial endothelial cells from renal allograft biopsy samples. ADPN co-localized with T-cadherin and AdipoR1, but only partially co-localized with AdipoR2.

**Conclusion:**

Both HMW-ADPN and HDL-C might inhibit the progression of vascular calcification by promoting ADPN binding to vascular endothelial cells via T-cadherin and AdipoR in Japanese renal allograft recipients.

## Introduction

The causes of chronic renal graft failure have been roughly classified into immunological and non-immunological mechanisms [1, 2], including dyslipidemia, which belongs to the latter type [3].

Furthermore, it was reported that the mortality rate due to cardiovascular disease is higher among renal allograft recipients than among non-renal allograft recipients, and it is considered that cardiovascular disease also influences renal allograft function [4]. Moreover, the components of metabolic syndrome, such as obesity, diabetes, hypertension, and dyslipidemia, are also important risk factors for cardiovascular complications. In addition, various kinds of pro-inflammatory cytokines and adipocytokines, which are secreted by adipose tissue, affect the risk of cardiovascular disease [5].

Adiponectin (ADPN) is mainly produced and subsequently secreted by the adipocytes in white and brown adipose tissue. It has anti-diabetes and anti-atherosclerotic effects and has an important influence on the conditions of patients with cardiovascular complications [6]. ADPN is found at high concentrations of 5–10 μg/ml in normal human blood and has a multimeric structure; i.e., it comes in high-molecular-weight (HMW-ADPN), middle-molecular-weight (MMW-ADPN), and low-molecular-weight (LMW-ADPN) forms [7]. Regarding the various ADPN fractions, a previous study reported that its HMW dodecamer and 18-mer, but not its monomer or trimer, were closely associated with the prevention of coronary artery disease, weight loss, and improved insulin resistance [8, 9].

On the other hand, the LMW-ADPN and MMW-ADPN fractions can pass through the blood-brain barrier and activate adenosine monophosphate (AMP)-activated protein kinase (AMPK) via adiponectin receptor 1 (AdipoR1) in the hypothalamus, which increases food intake and reduces energy consumption and fat accumulation in adipose tissue [10]. These findings indicate that LMW- and MMW-ADPN have opposite effects on weight to HMW-ADPN. In addition, an inverse correlation has been detected between the serum ADPN level and visceral fat area (VFA), but not subcutaneous fat area [11]. Furthermore, many studies of serum ADPN levels have revealed that high serum ADPN levels are associated with a reduced risk of diabetes [12], whereas reduced serum ADPN levels are associated with increases in VFA, the risk of cardiovascular disease [13], and atherosclerosis combined with dyslipidemia [14, 15], in both sexes. However, few long-term studies have been conducted on the correlations between vascular calcification and immunosuppressive drug use, lipid marker levels, including the concentrations of each ADPN fraction, in renal allograft recipients.

As for the mechanism responsible for the anti-atherosclerotic effects of circulating ADPN, ADPN regulates the oxyradical activity of vascular endothelial cells and activates endothelial nitric oxide synthase synthesis through AMPK [8, 16].

As for the mechanism by which ADPN binds to the vascular endothelium, AdipoR1 and R2 [17, 18] and T-cadherin [19–22] have been reported to be involved, but this remains to be confirmed in transplanted human kidneys.

In the present study, we investigated the serum levels of lipid markers, including each ADPN fraction, and their relationship with vascular calcification to evaluate the usefulness of HMW-ADPN as a predictor of cardiovascular complications in renal allograft recipients.

## Subjects and methods

### I. Subjects

Our subjects comprised 51 patients (34 males and 17 females) who had undergone renal transplants at Kanazawa Medical University Hospital and exhibited serum creatinine levels of ≤3 mg/dL, and in whom the transplant had engrafted by the start of the study period in 2008 and engraftment persisted until 2016.

The following factors were investigated: the age at transplant; sex; donor type (living or deceased renal donor); the human leukocyte antigen (HLA) concordance rate; the time since the transplant; the estimated glomerular filtration rate (eGFR) at the start of the study period; body mass index (BMI); blood pressure; the serum levels of total cholesterol (T chol), high-density lipoprotein cholesterol (HDL-C), low-density lipoprotein cholesterol (LDL-C), non-HDL-C (= T chol—HDL-C), and each ADPN fraction (the HMW- and non-HMW (= MMW + LMW)-ADPN fractions); immunosuppressive drug use; anti-diabetic drug (including insulin) use; statin use; and anti-hypertensive drug use.

We evaluated 1) the serum levels of lipid markers and each ADPN fraction, the renal function of the transplanted organs, and the aortic calcification area index (ACAI); 2) the correlations between serum levels of each ADPN fraction and vascular calcification; and 3) the correlations between the serum levels of each ADPN fraction or lipid markers and allograft function (eGFR) at the start and end of the study period.

Our study did not include any vulnerable populations, such as prisoners, subjects with reduced mental capacity due to illness or age, or children. In addition, we only used blood samples, radiological scans and renal biopsy specimens in this study. The study protocol was approved by the ethics committee of Kanazawa Medical University (Kanazawa Medical University Epidemiological Study Review No. 1127). All patients provided written informed consent, and the study was conducted according to the principles of the Declaration of Helsinki and Istanbul.

### II. Measurement methods

Serum creatinine levels were analyzed using an enzymatic method, a Hitachi creatinine auto-analyzer (model 7170; Hitachi, Tokyo, Japan), and an enzyme solution (Preauto-SCrE-N; Daiichi Pure Chemicals Co., Tokyo, Japan). The serum levels of T chol, LDL-C, and HDL-C were measured by direct enzymatic assays using an automatic analyzer (Hitachi, Tokyo, Japan). The serum levels of the total, HMW-, MMW-, and LMW-ADPN fractions were measured using a sensitive enzyme-linked immunosorbent assay kit (SEKISUI MEDICAL Co., Tokyo, Japan). Renal function was evaluated based on the eGFR.

The eGFR (= 194 x SCr^-1.094^ x age^-0.287^ x 0.739 for females, ml/min/1.73m^2^) was calculated based on the patients’ serum creatinine (SCr) levels, as described previously [23].

We evaluated the calcification of the abdominal aorta using the ACAI. The ACAI was calculated based on assessments of computed tomography scans of the abdominal aortic wall (slice thickness: 5 mm or 10 mm) in the region of interest. Specifically, it was calculated by assessing the percentage of the aortic wall occupied by calcification on each slice and then dividing the sum of the percentage values for all slices by the number of slices (S1 Fig). These analyses were conducted using image analysis software (MITANI Co., Ltd., Fukui, Japan).

### III. Immunohistopathological examinations

Fresh tissue specimens, which were embedded in OCT compound and frozen in acetone-dry ice mixture, were cut at a thickness of 3 μm on a cryostat. The frozen sections were fixed in 1:1 of acetone and methanol, and then blocked with 10% goat serum in 0.01 mol/L phosphate-buffered saline.

Staining of CD31, ADPN, AdipoR1, AdipoR2, and T-cadherin (cadherin-13) was performed by indirect immunofluorescence using the primary monoclonal or polyclonal antibodies listed in S1 Table.

Anti-mouse, rabbit, or goat IgG polyclonal antibodies conjugated with fluorescein isothiocyanate (MP Biomedicals), Alexa Fluor 455, or Alexa Fluor 488 (Thermo Fisher Scientific) were used as secondary antibodies, and their signals were visualized using a BX51/DP71 fluorescence microscope/CCD camera (Olympus).

### IV. Statistical analysis

All continuous variables are presented as median and interquartile range (IQR) values. The Mann-Whitney test was used for comparisons between the sexes and of the change in the ACAI. The changes of serum calcium (Ca), phosphate (p) and Ca x P product were evaluated by paired Student’s t-analysis. The relationships between the serum levels of lipid markers and the serum levels of each ADPN fraction were evaluated using Spearman’s correlation coefficient. The factors influencing the HMW-ADPN level, the relative HMW-ADPN (%) level compared with the non-HMW-ADPN level, and the ACAI were analyzed using multivariate regression analysis and the stepwise method. Stat Flex version 6 (Artech Co., Ltd., Osaka, Japan) was used as the statistical analysis software.

## Results

### I. Clinical background

Fifty-one renal allograft recipients (34 males and 17 females, 44 living donors and 7 deceased donors; Table 1) were enrolled in this study. Their median systolic and diastolic blood pressure values were 124 (111–130) and 80 (70–85) mmHg, respectively, and were relatively stable, with 48 (94%) subjects meeting the target blood pressure (systolic blood pressure of <140 mmHg). Regarding cholesterol, the serum LDL-C level was <120 mg/dL in 37 (73%) patients, while the serum HDL-C level was ≥40 mg/dL in 47 (92%) patients. The serum level of triglycerides was <150 mg/dL in 34 (67%) patients. Thirty-five subjects (69%) were being treated with pravastatin or atorvastatin. The females exhibited much higher serum phosphate levels than the males (p<0.05). No significant intersex difference was noted in the number of HLA mismatches or the eGFR.

**Table 1:** The subjects’ baseline characteristics.

Variable	Total	Males	Females
N	51	34	17
Donor (living: deceased)	44: 7	30: 4	14: 3
Age at transplant, years	31.0 (24.0–35.8)	32.5 (24.0–36.0)	30.0 (24.3–35.0)
Duration of dialysis, years	16.2 (8.4–50.1)	15.5 (8.5–50.3)	21.2 (8.2–61.3)
Time since Tx (months)	286 (219–371)	296 (241–384)	233 (203–334)
BMI	21.0 (19.2–22.3)	21.0 (19.4–23.2)	20.6 (18.6–21.7)
Blood pressure (mmHg)			
Systolic	124 (111–130)	121 (112–130)	130 (110–133)
Diastolic	80 (70–85)	74 (70–80)	80 (72–88)
eGFR in 2008 (ml/min)	51.5 (42.4–58.8)	53.6 (43.2–65.4)	48.3 (39.3–56.3)
eGFR in 2016 (ml/min)	47.7 (39.7–58.6)	52.8 (41.3–59.5)	42.8 (38.1–55.4)
ΔeGFR (ml/min)	-0.25 (-0.82–0.35)	-0.25 (-0.58–0.4)	-0.2 (-0.99–0.23)
Serum Ca (mg/dl)	9.6 (9.3–9.8)	9.7 (9.4–9.9)	9.5 (9.2–9.6)
Serum phosphorus (mg/dl)	2.9 (2.6–3.2)	2.8 (2.4–3.1)	3.1 (2.9–3.4)*
LDL-C (mg/dL)	103.0 (85.3–122.5)	112.0 (97.0–132)**	90.0 (76.5–98.3)
HDL-C (mg/dL)	61.0 (54.3–78.8)	56.5 (51.0–66.0)	78.0 (66.8–96.5)**
LDL-C/HDL-C ratio	1.68 (1.18–2.21)	1.98 (1.52–2.53)**	1.15 (0.92–1.41)
TG (mg/dl)	127 (93.3–174.8)	130 (109–184)	116 (85–169)
non-HDL-C (mg/dl)	128.6 (110.7–153.8)	144.3 (119.6–167)**	111.4 (97.7–131.5)
Blood glucose (mg/dl)	95 (86.3–108.3)	97 (87–120)	92 (81–99)
Total ADPN in 2008 (μg/ml)	7.5 (5.4–10.2)	6.7 (5.0–8.4)	10.2 (7.4–11.4)*
HMW-ADPN (μg/ml)	2.7 (1.7–4.5)	2.5 (1.7–3.6)	4.5 (3.0–6.8)**
MMW-ADPN (μg/ml)	1.7 (1.2–2.3)	1.7 (1.2–1.9)	2.3 (1.8–2.8)**
LMW-ADPN (μg/ml)	2.6 (2.1–3.3)	2.5 (2.2–3.3)	2.9 (2.0–3.3)
Relative HMW-ADPN (%)	39.4 (31.5–45.2)	35.0 (29.0–42.0)	45.0 (37.4–55.6)**
Relative MMW-ADPN (%)	22.4 (20.2–27.1)	23.3 (20.3–27.0)	20.9 (20.1–27.8)
Relative LMW-ADPN (%)	38.1 (30.1–45.9)	42.7 (32–46.5)**	29.6 (21.4–37.4)
ACAI	0.08 (0.00–1.28)	0.15 (0.00–1.41)	0.00 (0.00–1.26)
Therapeutic agents (drug use, %)			
Immunosuppressive drugs			
Steroids	51 (100%)	34 (100%)	17 (100%)
Anti-metabolites	49 (96%)	33 (97%)	16 (94%)
Calcineurin inhibitors	36 (71%)	24 (71%)	12 (77%)
Anti-hypertensive drugs	38 (75%)	30 (88%)	8 (65%)
Anti-diabetic drugs	4 (8%)	3 (9%)	1 (6%)
Insulin	2 (4%)	2 (6%)	0 (0%)
Oral anti-diabetic drugs	2 (4%)	1 (3%)	1 (6%)
Statins	35 (69%)	24 (71%)	11 (65%)
Bisphosphonates	9 (18%)	5 (15%)	4 (24%)

Abbreviations: Tx, transplant; eGFR, estimated glomerular filtration rate; LDL-C, low-density lipoprotein cholesterol; HDL-C, high-density lipoprotein cholesterol; ADPN, adiponectin; HMW, high molecular weight; MMW, middle molecular weight; LMW, low molecular weight; ACAI, aortic calcification area index; TG, triglycerides. One patient was complicated by a cerebral infarction and angina pectoris. Data are shown as median (IQR) values.

*<0.05

**<0.01, according to the Mann-Whitney test

The serum levels of LDL-C and non-HDL-C were higher in males (p<0.05 and p<0.01, respectively), while the serum levels of HDL-C and total, HMW-, and MMW-ADPN and the relative HMW-ADPN level were higher in females (p<0.01). Although no significant intersex difference in the serum LMW-ADPN level was detected, the relative LMW-ADPN level was higher in males. No significant intersex differences in the ACAI or drug use were observed.

### II. Relationships between the eGFR and serum ADPN levels in renal graft recipients

The relationships between renal allograft function and the serum levels of each ADPN fraction are shown in S2 Fig. Both the HMW- and non-HMW-ADPN levels were inversely correlated with the eGFR ([A] r = -0.300, p<0.032, n = 51 and [B] r = -0.385, p<0.005, n = 51, respectively), whereas the relative HMW-ADPN level was not significantly correlated with the eGFR (r = -0.233, p = 0.116, n = 51, [C]).

### III. Relationships between the serum HMW-ADPN level and the serum levels of each lipid marker

The associations between the serum HMW-ADPN level and the serum levels of lipid markers at the start of the study period are shown in S2 Table. The relative HMW-ADPN level was negatively correlated with the serum levels of LDL-C, triglycerides (TG), and non-HDL-C (r = -0.324, p = 0.02; r = -0.291, p = 0.038; and r = -0.357, p = 0.01; respectively). In addition, the relative HMW-ADPN level was positively correlated with the serum HDL-C level (r = 0.314, p = 0.025). Moreover, the serum HDL-C level was negatively correlated with BMI in 2008 (r = -0.379, p = 0.006).

### IV. The changes in renal function, the serum levels of lipid markers and ADPN, and the ACAI during the 8-year study period in renal allograft recipients

Renal allograft function, the serum levels of lipid markers and each ADPN fraction, and the ACAI at the start (2008) and end (2016) of the study period, and the median annual changes in each parameter are shown in Table 2.

**Table 2:** The changes in renal function, the ACAI, and the serum levels of lipid markers and ADPN during the 8-year study period in renal allograft recipients.

	2008	2016	Δ	p
eGFR (ml/min)	51.3 (42.5–58.2)	48.2 (40.0–58.4)	-0.25 (-0.82–0.35)	0.0375*
LDL-C (mg/dL)	103.0 (85.3–122.5)	105.0 (82.3–127.3)	0.38 (-1.34–2.03)	0.3770 *
TG (mg/dL)	127 (93.3–174.8)	133.0 (110.1–185.9)	1.00 (-0.40–5.5)	0.3201 *
non-HDL-C (mg/dL)	128.6 (110.7–153.8)	135.0 (107.8–163.9)	0.90 (-2.29–3.25)	0.3732 *
HDL-C (mg/dL)	61.0 (54.3–78.8)	59.0 (50.0–73.8)	-0.13 (-0.97–0.47)	0.1450 *
HMW-ADPN	2.7 (1.7–4.5)	3.3 (1.8–5.8)	0.02 (-0.06–0.15)	0.0712 *
non-HMW-ADPN	4.6 (3.7–5.7)	4.1 (3.1–5.3)	-0.03 (-0.11–0.07)	0.2567 *
Relative HMW-ADPN (%)	39.4 (31.5–45.2)	45.1 (32.1–52.3)	0.47 (-0.21–1.02)	0.0044 *
Relative non-HMW-ADPN (%)	60.6 (54.8–68.5)	54.9 (47.7–67.9)	-0.46 (-1.03–0.22)	0.0047 *
ACAI	0.08 (0.00–1.28)	1.67 (0.08–4.73)	0.17 (0.004–0.42)	<0.001 *
Calcium (Ca) (mg/dL)	9.60 (9.30–9.80)	9.80 (9.60–10.00)	0.20 (-0.10–0.50)	0.052 **
Phosphate (P) (mg/dL)	2.90 (2.60–3.20)	2.80 (2.50–3.30)	0.00 (-0.40–0.40)	0.862 **
Ca x P product	28.00 (25.11–30.38)	27.84 (24.50–31.68)	0.71 (-3.73–4.67)	0.736 **

Abbreviations: eGFR, estimated glomerular filtration rate; LDL-C, low-density lipoprotein cholesterol; HDL-C, high-density lipoprotein cholesterol; HMW-ADPN, high-molecular-weight adiponectin; ACAI, aortic calcification area index; TG, triglycerides. One patient was complicated by a cerebral infarction and angina pectoris. Data are shown as median (IQR) values. P values were evaluated by

(*) Mann-Whitney test or

(**) paired- Student’s t test

The eGFR, a measure of renal allograft function, had decreased at the end of the 8-year study period (p = 0.037). On the other hand, there were no significant changes in the serum levels of LDL-C, TG, non-HDL-C, HDL-C, HMW-ADPN, or non-HMW-ADPN. The relative HMW-ADPN level increased significantly from 39.4 to 45.1% during the 8-year study period (p = 0.0044). On the other hand, abdominal aorta calcification (the ACAI) increased significantly from 0.08 to 1.67 (p<0.001).

Furthermore, in the multivariate regression analysis the serum HDL-C level was identified as a factor that influenced the annual change in the serum HMW-ADPN level (beta value: 0.078, t value: 3.091, p = 0.004 in model 1, beta value: 0.067, t value: 2.815, p = 0.007 in model 2; Table 3).

**Table 3:** The factors that influenced the changes in the HMW-ADPN level and the ACAI in renal allograft recipients.

Objective variable: Change in the HMW-ADPN level					
Model 1	β	SE	Std β	t	p
(Constant)	0.280	0.111			
Change in HDL-C	0.078	0.025	0.463	3.091	0.004
Change in non-HDL-C	-0.003	0.0054	-0.078	0.575	0.568
Change in BMI	-0.132	0.104	-0.170	1.276	0.209
Age at transplant	-0.005	0.004	-0.192	1.318	0.195
Cardiovascular disease	-0.030	0.103	-0.046	0.293	0.771
Post-transplant DM	-0.009	0.075	-0.021	0.122	0.904
Objective variable: Change in the HMW-ADPN level					
Model 2	β	SE	Std β	t	p
(Constant)	0.225	0.105			
Change in HDL-C	0.067	0.024	0.398	2.815	0.007
Change in LDL-C	0.002	0.006	0.050	0.366	0.716
Change in BMI	-0.128	0.105	-0.165	1.217	0.230
Age at transplant	-0.005	0.004	-0.199	1.350	0.184
Cardiovascular disease	0.002	0.101	0.004	0.0231	0.982
Post-transplant DM	-0.038	0.074	-0.086	0.511	0.612

Abbreviations: LDL-C, low-density lipoprotein cholesterol; HDL-C, high-density lipoprotein cholesterol; BMI, body mass index; DM, diabetes mellitus. Multiple logistic regression models were developed to identify predictors of the HMW-ADPN level and the ACAI in a step-wise manner. Explanatory variables: *Model 1*: age at transplant, gender, statin use, cardiovascular disease, post-transplant DM, change in eGFR, change in HDL-C level, change in non-HDL-C level, change in BMI. *Model 2*: age at transplant, gender, statin use, cardiovascular disease, post-transplant DM, change in eGFR, change in HDL-C level, change in LDL-C level, change in BMI

### V. The factors that influenced the change in the ACAI in renal allograft recipients

**Fig 1:**
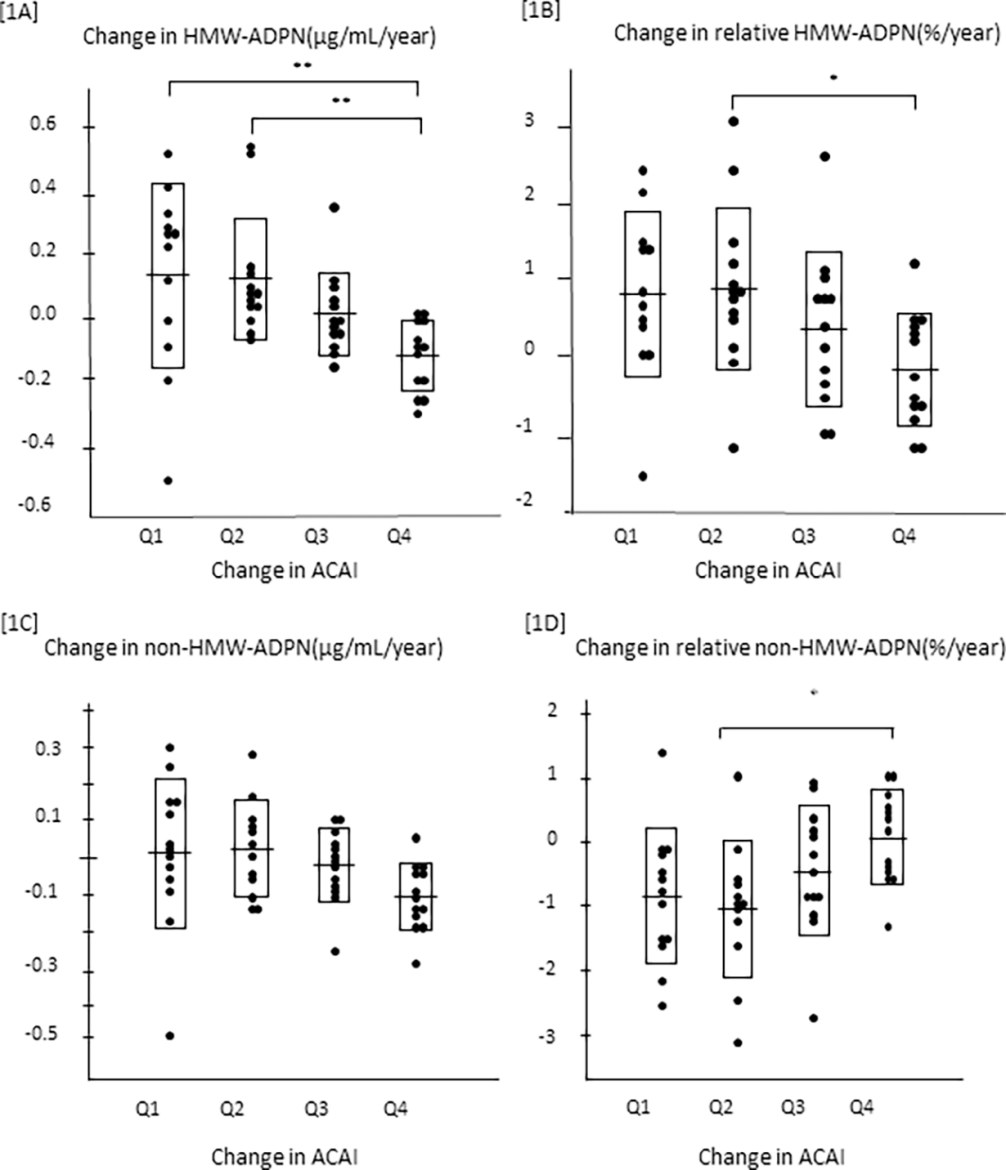
The influence of the serum HMW-ADPN level on the change in the ACAI in renal allograft recipients. The annual change in the ACAI was divided into 4 quartiles (Q1, <0.006, n = 13; Q2, 0.006–0.172, n = 12; Q3, 0.172–0.413, n = 13; Q4, >0.413, n = 13). The annual changes in both the absolute [1A] and relative [1B] serum HMW-ADPN levels became smaller as abdominal aortic calcification (ACAI) progressed (p<0.01, according to Dunn’s test). The annual change in the serum non-HMW-ADPN level [1C] also tended to decline as the ACAI increased. On the contrary, the annual change in the relative serum non-HMW-ADPN level [1D] significantly increased as the ACAI rose (p<0.05, according to Dunn’s test).

The annual change in the ACAI was divided into 4 quartiles (Q1, <0.006, n = 13; Q2, 0.006–0.172, n = 12; Q3, 0.172–0.413, n = 13; Q4, >0.413, n = 13;Fig 1).

Both the annual changes in the absolute [A] and relative [B] HMW-ADPN levels got smaller as the abdominal aortic calcification (the ACAI) progressed (p<0.01, according to Dunn’s test). The annual change in the non-HMW-ADPN level [C] also tended to become smaller as the abdominal aortic calcification progressed. On the contrary, the annual change in the relative non-HMW-ADPN level [D] increased significantly as the ACAI rose (p<0.05, according to Dunn’s test).

Furthermore, the annual change in the serum HMW-ADPN level, a history of cardiovascular disease, and age at renal transplant were identified as factors that had a significant influence on the annual change in the ACAI in the multivariate regression analysis (beta value: -0.102, t value: 2.671, p = 0.010 for HMW-ADPN; beta value: 0.380, t value: 2.749, p = 0.008 for a history of cardiovascular disease; beta value: 0.017, t value: 3.706, p<0.001 for age; Table 4).

**Table 4:** The factors that influenced the changes in the ACAI in renal allograft recipients.

Objective variable: Change in the ACAI					
	Β	SE	Std β	t	p
(Constant)	-0.212	0.148			
Age at transplant	0.017	0.004	0.468	3.706	<0.001
Cardiovascular disease	0.380	0.138	0.368	2.749	0.008
Change in HMW-ADPN	-0.102	0.038	-0.312	2.671	0.010
Post-transplant DM	-0.193	0.100	-2.777	1.921	0.061
Change in eGFR	-0.078	0.044	-0.202	1.746	0.087
Statin use	-0.058	0.085	-0.081	-0.685	0.498
Change in Ca x P product	0.002	0.007	0.046	0.379	0.707
Dialysis period before RTx	0.001	0.001	0.104	0.836	0.408

Abbreviations: LDL-C, low-density lipoprotein cholesterol; HDL-C, high-density lipoprotein cholesterol; BMI, body mass index; DM, diabetes mellitus; RTx, renal transplantation. Multiple logistic regression models were developed to identify predictors of the HMW-ADPN level and the ACAI in a step-wise manner. Explanatory variables: age at transplant, gender, dialysis period before renal transplantation, statin use, cardiovascular disease, post-transplant DM, change in the eGFR, change in the HDL-C level, change in the LDL-C level, change in BMI, and change in the HMW-ADPN level, change in Ca x P product

### VI. Renal expression of ADPN, AdipoR1, AdipoR2, and T-cadherin

**Fig 2:**
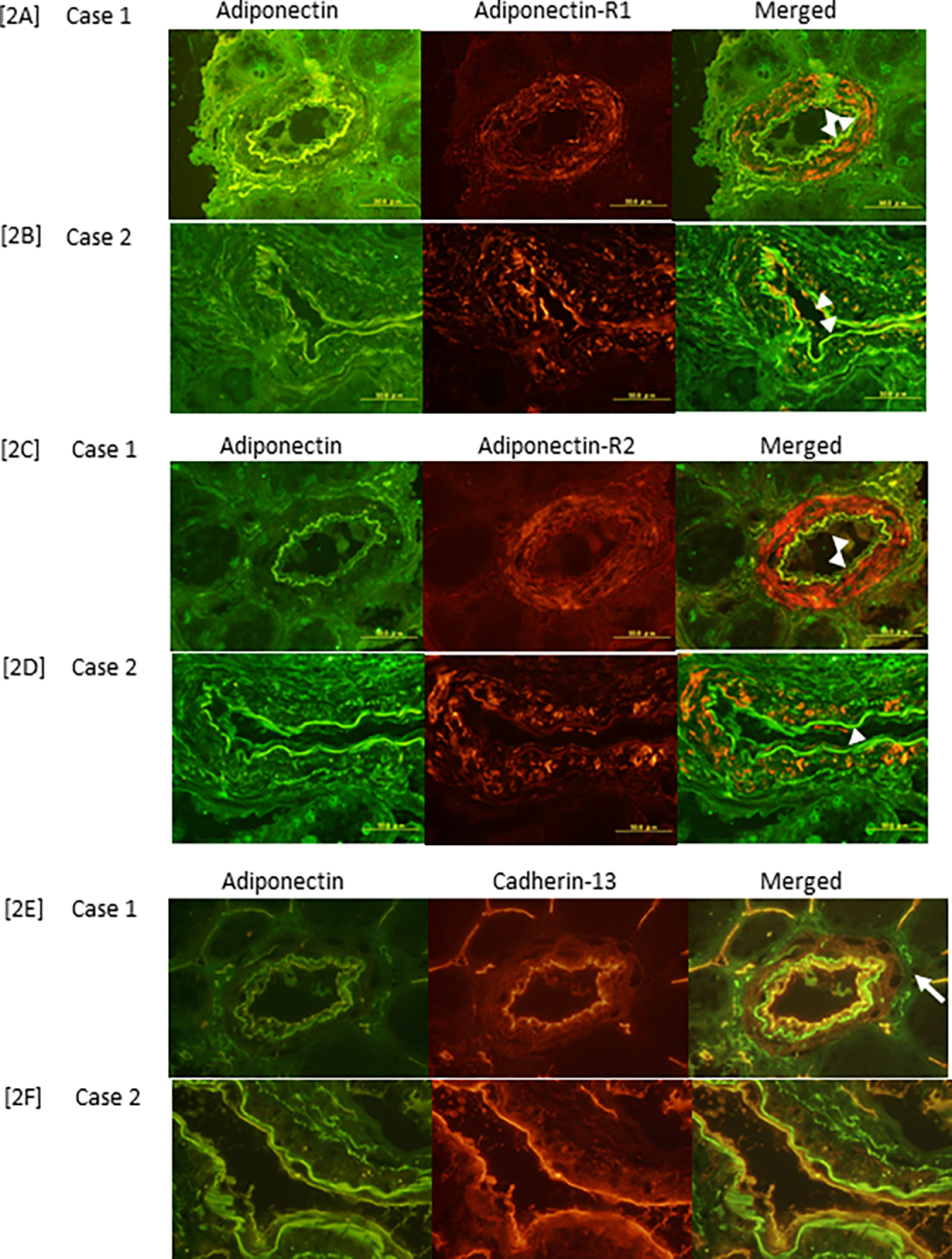
ADPN, AdipoR1/R2 and cadherin-13 (T-cadherin) expression in renal allografts. ADPN and AdipoR1 co-localized on the arterial endothelium (arrow) in cases 1 [2A] and 2 [2B]. The endothelium exhibited weaker AdipoR2 expression (arrow) than AdipoR1 expression in cases 1 [2C] and 2 [2D]. ADPN and cadherin-13 co-localized linearly on vascular endothelial cells (arrow) in cases 1 [2E] and 2 [2F].

In the renal allografts, ADPN was linearly detected along the CD31-positive endothelia of the middle-sized muscular arteries during immunohistochemical examinations (S3 Fig). ADPN co-localized with AdipoR1 and partially co-localized with AdipoR2 on the arterial endothelia. On the other hand, cadherin-13 (T-cadherin) exhibited a linear expression pattern on the inner vascular walls of the arteries and the endothelial cells of the peritubular capillaries, and was recognized to co-localize with ADPN (Fig 2).

## Discussion

We obtained three important results in this study. The first was that the serum HMW-ADPN level was positively correlated with the serum HDL-C level; i.e., an increase in the HDL-C level during the follow-up period was associated with a rise in the HMW-ADPN level, in renal allograft recipients. The second was that calcification of the abdominal aorta might be suppressed by an increase in the relative serum level of HMW-ADPN. The third was that ADPN mainly co-localized with T-cadherin and AdipoR1 on the endothelia of arteries in transplanted kidneys.

Relationships between the serum ADPN level, especially the serum HMW-ADPN level, and renal function have been reported to exist in many types of chronic kidney disease (CKD) [24]. Similarly, we previously reported that a negative correlation exists between the eGFR and the serum HMW-ADPN level in a cross-sectional study of renal allograft recipients [25]. Furthermore, the so-called “adiponectin paradox”, which states that higher serum ADPN levels are associated with lower eGFR, was reported in studies of chronic renal failure patients [26, 27].

The current study also detected negative correlations between renal function (eGFR) and the serum levels of both HMW-ADPN and non-HMW-ADPN, but not the relative serum levels of these substances. Otherwise, there were no other parameters that were significantly associated with either the serum HMW-ADPN level or the serum non-HMW-ADPN level, but the serum HMW-ADPN level increased during the follow-up period in renal allograft recipients. Thus, it was suggested that not only the serum ADPN level, but also the serum levels of each ADPN fraction, are affected by renal function.

Cardiovascular disease, which is the main cause of death in renal allograft recipients, is strongly associated with atherosclerotic lesions [28]. In addition, the risk of a cardiovascular event was reported to be 3.5–5% in renal allograft recipients, which is 50 times higher than that seen in the normal population [29]. Moreover, the incidence of atherosclerosis-related cardiovascular events, hypertension, and dyslipidemia were found to increase when the serum ADPN level fell [30]. Our previous study also revealed that an increase in the relative HMW-ADPN level and the use of statins reduced the risk of cardiovascular events after renal transplant, whereas an increased LDL-C level was found to be associated with a higher risk of cardiovascular events, even in renal allograft recipients [25].

It was reported that the annual growth rate of atheromas in the carotid arteries plateaus around the G3 stage in CKD patients [31]. Furthermore, the risk of atherosclerosis-related cardiovascular disease increases after CKD progresses to stage G3-G4. After the progression of CKD to stage G4-5, non-atherosclerotic disease becomes the leading risk factor for cardiovascular disease [32]. In this study, the relative serum HMW-ADPN level was found to be positively correlated with the serum HDL-C level, but negatively correlated with the serum levels of LDL-C and non-HDL-C. In addition, the patients with advanced calcification of the abdominal aorta displayed lower absolute and relative serum HMW-ADPN levels. Our results suggest that treating dyslipidemia might ameliorate arterial calcification by increasing the serum HMW-ADPN level in renal allograft recipients because the patients’ renal function improved from CKD stage G5D to stage G2-G3aT after the renal transplants. Otherwise, a recent report by Chen et al. found that repeated coronary artery calcification (CAC) imaging in 35 patients with end-stage renal disease showed that statin therapy was associated with greater progression of CAC. They also found that in vitro synthesis of menaquinone-4 by human vascular smooth muscle cells was significantly impaired by statins [33]. Hence, novel therapies to target lipids may be needed in patients with kidney disease in future.

With respect to the molecular mechanisms underlying the effects of ADPN, they have been reported to involve the binding of ADPN with its receptors and T-cadherin [17–22]. In particular, AdipoR1 and AdipoR2 have been shown to be the major ADPN receptors in vivo [17]. AdipoR1 is mainly distributed in skeletal muscles and promotes the uptake of glucose and the consumption of fatty acids through the activation of AMPK [18]. On the other hand, AdipoR2 is most abundantly expressed in the liver, and the insulin-sensitizing effects of ADPN were shown to be mediated via the activation of the peroxisome proliferator-activated receptor γ (PPARγ) [17]. Moreover, both AdipoR1 and AdipoR2 were found in blood vessels and macrophages [18].

T-cadherin is capable of binding to ADPN, although T-cadherin itself is thought to have no effect on the cellular signaling or functions of ADPN because it lacks an intracellular domain [30]. T-cadherin expression on vascular endothelia, smooth muscle cells, and cardiomyocytes might aid the binding of ADPN to AdipoR1/R2 in the heart [19]. In addition, circulating ADPN can bind to ADPN receptors after first binding to T-cadherin [20]. Furthermore, in atherosclerotic lesions ADPN accumulated in vascular endothelial cells and synthetic type smooth muscle cells, and protected vascular functions by binding to T-cadherin [21, 22].

In this study, we examined the associations between ADPN expression and AdipoR1/R2 or T-cadherin expression in human renal allograft biopsy specimens. Linear ADPN expression was observed on the vascular endothelia of the renal specimens. Furthermore, AdipoR1 expression was predominantly found on the endothelia of medium-sized muscular arteries. ADPN and AdipoR1 co-localized linearly in parts of the arterial endothelia. On the other hand, AdipoR2 was predominantly detected on the smooth muscle cells of the medial arterial layer, and it co-localized with ADPN in small areas of the arterial endothelia. It was suggested that ADPN also binds to molecules other than its receptors on vascular endothelia because while continuous linear ADPN expression was detected on vascular endothelia the expression of AdipoR1 on arterial endothelia was often discontinuous. We observed the linear co-localization of ADPN and T-cadherin in both arterial and capillary endothelial cells. These findings suggest that T-cadherin might act as an adhesion molecule for ADPN in humans, as was found in a previous mouse study [21].

## Limitations

This study had the following limitations: 1) The small number of subjects meant that the study was at risk of bias due to its population (a Japanese population). 2) As the study was retrospective in nature, the use of drugs such as statins was not uniform. 3) Only a few transplanted kidneys were examined by immunohistochemistry. Therefore, it will be necessary to perform a long-term prospective clinical study in future.

## Conclusion

The positive association between the levels of HMW-ADPN and HDL-C might inhibit the progression of vascular calcification by promoting ADPN binding to vascular endothelial cells via T-cadherin and AdipoR in Japanese renal allograft recipients.

## Supporting information

S1 Fig The ACAI was calculated based on assessments of computed tomography scans of the abdominal aortic wall (slice thickness: 5 mm or 10 mm) in the region of interest. Specifically, it was calculated by assessing the percentage of the aortic wall occupied(A/B) by calcification on each slice and then dividing the sum of the percentage values for all slices by the number of slices.
(TIF)

S2 Fig Both the HMW- and non-HMW-ADPN levels were inversely correlated with the eGFR ([A] r = -0.300, p<0.032, n = 51 and [B] r = -0.385, p<0.005, n = 51, respectively), whereas the relative HMW-ADPN level was not significantly correlated with the eGFR (r = -0.233, p = 0.116, n = 51, [C]).
(TIF)

S3 Fig ADPN was linearly detected along the CD31-positive endothelia of the middle-sized muscular arteries during immunohistochemical examinations. (TIF)

S1 Table The species, dilution values, and sources of the primary and secondary antibodies. (DOCX)

S2 Table The correlation between the serum adiponectin level and the concentration of each lipid marker. (DOCX)
