# Supplementary figures and images for: High molecular weight adiponectin inhibits vascular calcification in renal allograft recipients
Source: PLoS One. 2018 May 2;13(5):e0195066. doi: 10.1371/journal.pone.0195066 (PMC5931493; doi:10.1371/journal.pone.0195066)

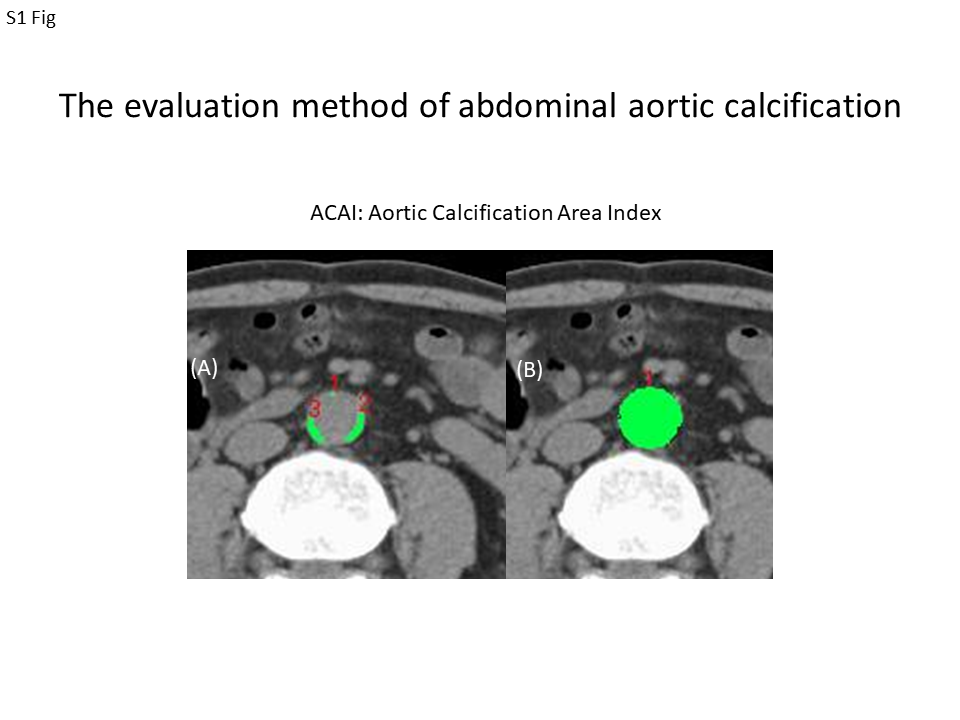

Supplement: S1 Fig — Specifically, it was calculated by assessing the percentage of the aortic wall occupied(A/B) by calcification on each slice and then dividing the sum of the percentage values for all slices by the number of slices. (TIF) [file pone.0195066.s001.tif]

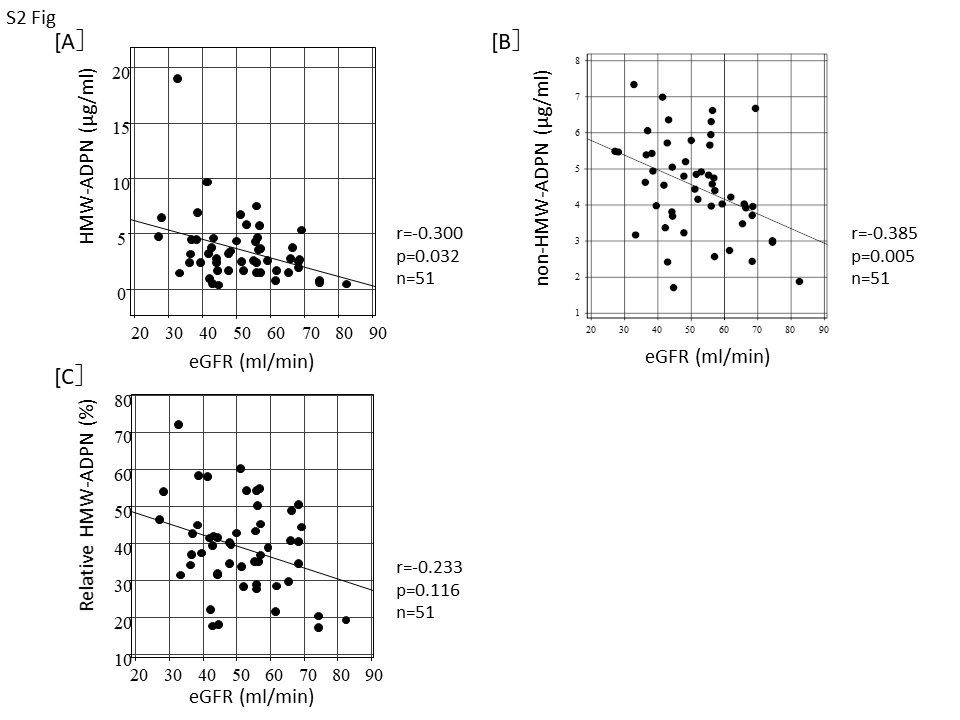

Supplement: S2 Fig — Both the HMW- and non-HMW-ADPN levels were inversely correlated with the eGFR ([A] r = -0.300, p<0.032, n = 51 and [B] r = -0.385, p<0.005, n = 51, respectively), whereas the relative HMW-ADPN level was not significantly correlated with the eGFR (r = -0.233, p = 0.116, n = 51, [C]). (TIF) [file pone.0195066.s002.tif]

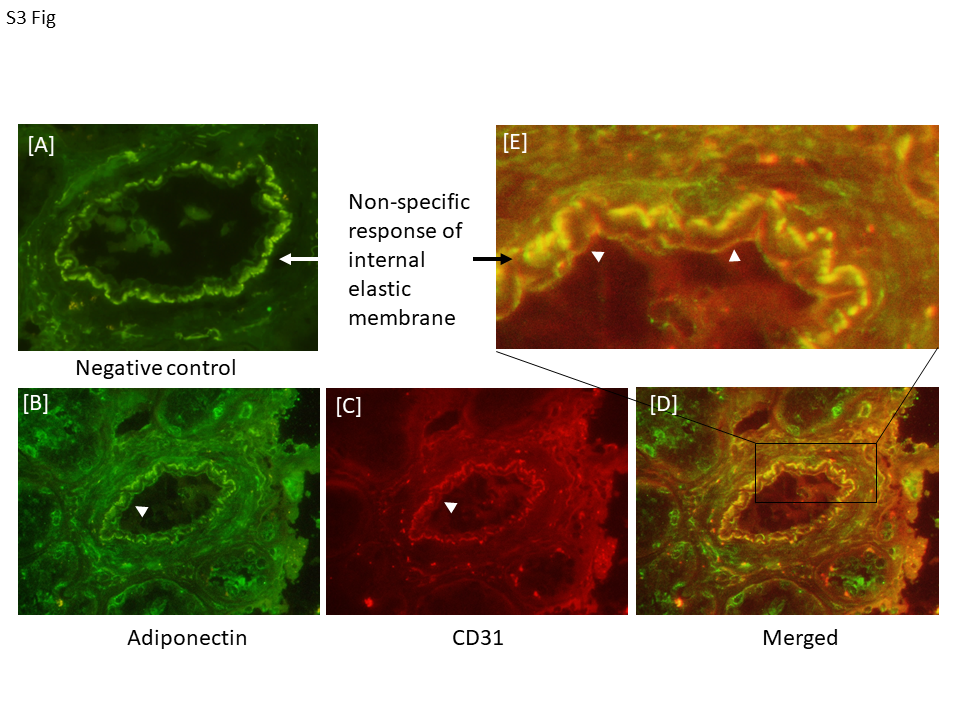

Supplement: S3 Fig — (TIF) [file pone.0195066.s003.tif]
