# Supplementary material for: High molecular weight adiponectin inhibits vascular calcification in renal allograft recipients
Source: PLoS One. 2018 May 2;13(5):e0195066. doi: 10.1371/journal.pone.0195066 (PMC5931493; doi:10.1371/journal.pone.0195066)
Supplement: S1 Table — (DOCX) [file pone.0195066.s004.docx]

**S1 Table. The species, dilution values, and sources of the primary and secondary antibodies**

| Primary antibody | Species | Dilution | Product No. | Source |
| --- | --- | --- | --- | --- |
| Adiponectin | Mouse anti-human monoclonal | 1:40 | ab22554 | Abcam, Cambridge,  UK |
| CD31 | Rabbit anti-human polyclonal | 1:20 | bs-0195R | Bioss, USA |
| AdipoR1 | Rabbit anti-human monoclonal | 1:50 | ab126611 | Abcam, Cambridge,  UK |
| AdipoR2 | Rabbit anti-human polyclonal | 1:50 | Ab189446 | Abcam, Cambridge,  UK |
| Cadherin-13 | Goat anti-human polyclonal | 1:20 | AF3264 | R&D Systems, USA & Canada |
|  |  |  |  |  |
| Secondary antibodies | Conjugate | Dilution | Product No. | Sources |
| Anti-mouse IgG | FITC | 1:200 | 55504 | MP Biomedicals, USA |
|  | Alexa Fluor 488 | 1:300 | A-11059 | Thermo Fisher  Scientific, USA |
| Anti-rabbit IgG | Alexa Fluor 488 | 1:300 | A-11034 | Thermo Fisher  Scientific, USA |
|  | Alexa Fluor 555 | 1:700 | A-21429 | Thermo Fisher  Scientific, USA |
| Anti-goat IgG | FITC | 1:400 | 55353 | MP Biomedicals, USA |
|  | TRITC | 1:40 | 55357 | MP Biomedicals, USA |

AdipoR1, adiponectin receptor 1; AdipoR2, adiponectin receptor 2; FITC, fluorescein isothiocyanate; TRITC, tetramethylrhodamine
