# Supplementary material for: High molecular weight adiponectin inhibits vascular calcification in renal allograft recipients
Source: PLoS One. 2018 May 2;13(5):e0195066. doi: 10.1371/journal.pone.0195066 (PMC5931493; doi:10.1371/journal.pone.0195066)
Supplement: S2 Table — (DOCX) [file pone.0195066.s005.docx]

**S2 Table. The correlation between the serum adiponectin level and the concentration of each lipid marker**

|  |  | r* | p |
| --- | --- | --- | --- |
| Relative HMW-ADPN | HDL-C | 0.314 | 0.025 |
| Relative HMW-ADPN | LDL-C | -0.324 | 0.020 |
| Relative HMW-ADPN | TG | -0.291 | 0.038 |
| Relative HMW-ADPN | non-HDL-C | -0.357 | 0.010 |
| Relative HMW-ADPN | BMI | -0.200 | 0.111 |
| HDL-C | BMI | -0.370 | 0.007 |

HMW-ADPN: high-molecular-weight adiponectin, HDL-C: high-density lipoprotein cholesterol, LDL-C: low-density lipoprotein cholesterol, TG: triglycerides, BMI: body mass index

*: according to Spearman’s rank correlation coefficient
